# ASReml-based estimation of early genetic parameters in hybrid mutton sheep populations

**DOI:** 10.1080/10495398.2024.2383261

**Published:** 2024-08-02

**Authors:** Haifeng Wang, Shanning Jin, Zhenfei Xu, Jinxia Zhang, Xuejiao An, Rui Zhang, Yanyan Hao, Lina Zhu, Yinchun Wang, Junni Jin, Zhiguang Geng, Chenglan Li, Jianye Li, Yaojing Yue

**Affiliations:** a Chinese Academy of Agricultural Sciences, Lanzhou Institute of Husbandry and Pharmaceutical Sciences, Lanzhou, China; b Huanxian Animal Husbandry Technology Extension Centre, Huanxian, China; c Qingyang Research Institute of Agricultural Sciences, Qingyang, China

**Keywords:** Hybrid mutton sheep, ASReml, genetic parameter estimation, non-genetic factors, growth curves, correction formula

## Abstract

The aim of this study was to analyze the effects of non-genetic factors on the estimation of genetic parameters of early growth traits in hybrid mutton sheep using ASReml software, in order to provide theoretical basis for screening the optimal hybriding combinations and accelerating the breeding process of new breeds of specialized housed-feeding mutton sheep. We selected the wellgrown hybrid Southhu (Southdown × Hu sheep) and Dorhu (Dorset × Hu sheep) sheep as the research objects, constructed weight correction formulae for SH and DH sheep at 60 and 180 days; and used ASReml software to investigate the effects of non-genetic factors on the estimation of genetic parameters of early growth traits in hybrid sheep. The results showed that the birth month and birth type were found significant for all traits (*p* < 0.001); the heritability of maternal effects ranged from 0.0709 to 0.1859. It was found that both SH and DH sheep emerged the potential for rapid early growth and development, early growth traits are significantly affected by maternal genetic effects, thereby the maternal effect should be taken into consideration for the purpose of improving accuracy in parameter estimations and therefore increasing the success of breeding programs.

## Introduction

As consumers increasingly pursuing balanced dietary nutrition, the demand for high-quality lamb is growing year by year, and China has become the world’s largest producer and consumer of sheep mutton.^1^ Fewer specialized mutton sheep breeds with poor performance has become a lethal issue that constrained the development of the mutton sheep industry. Hu sheep, a unique domestic sheep germplasm resource in China, which is known for its excellent characteristics, including perennial estrus, high fecundity, novel lactation performance and strong adaptability, is an important dam breed in commercial hybrid mutton sheep.^2^ Southdown and Dorset are famous sheep breeds with perfect mutton production performance, which show the advantages of early growth, excellent mutton quality, easy to fatten and other characteristics, are considered to be the most popular breeds in mutton production, so as to usually utilized as the sire breeds in hybrid mutton sheep.^3^^,^^4^ Therefore, we carried out economic hybriding with local Hu sheep as the female parent, Southdown and Dorset as the sire to produce Southhu (Southdown × Hu sheep, SH) and Dorhu (Dorset × Hu sheep, DH) sheep.

In order to obtain more reliable and accurate genetic parameters for early growth traits in hybrid mutton sheep, it is necessary to determine the non-genetic factors affecting the accurate estimation of genetic parameters in advance, and then implement the accurate estimation of the genetic parameters for each trait, because it is not possible to measure the performance of each sheep according to the set ideal time in production. Therefore, in the present study, we proposed to use Gompertz,^5^ Logistic,^6^ and Von Bertalanffy^7^ nonlinear growth models to fit the optimal growth curves for the selection of DH and SH sheep, followed by constructing correction equations for body weights at 60 days as well as at 180 days of DH and SH sheep, and subsequently explored the effects of different nongenetic factors on the early growth traits (birth weight, weaning weight, and 6-month weight) of DH and SH sheep by using of the ASReml software. The effects of different non-genetic factors on the estimation of genetic parameters for early growth traits of DH and SH sheep were analyzed by ASReml software, also. Our study lays the foundation for screening optimal hybriding combinations and accelerating the breeding of new breeds of specialized housed-feeding mutton sheep.

## Materials and methods

### Experimental animals

This study was conducted at Gansu Qinghuan Mutton Sheep Breeding Co. Healthy lambs born in 2021 ∼ 2023 were selected as the experimental animals, and the daily nutrition supply standard of each sheep was referred to the feeding standard of Chinese mutton sheep, that fed with the same Total Mixed Ration (TMR) and free water. The experimental sheep were all housed and fed on a regular basis, disinfected, vaccinated and dewormed.

### Measurement methods and data processing

Birth weight were determined and recorded within 24 hours of birth. At 60 days, the weaned sheep were divided and weighted. After that, we weighted every 30 days and entered into a flock management system. Notably, weighing should be achieved before feeding and watering in the morning. The data were preliminarily organized and screened by Excel, in which the clearly genealogic, complete recorded and accurate information were utilized for the following study. Next, Logistic, Gompertz, and Von Bertalanffy models were used to determine the optimal fitting model by the degree of fit; one-way linear regression analyses were carried out for different growth stages, and the correction formulas for body weights at 60 and 180 days were constructed according to the linear regression equations obtained, and the differences between correction formulas of different breeds were examined to determine the scope of application of the correction formulas for body weights. Birth weight (BWT), weaning weight (WWT) and six months weight (SMW) were selected for early growth traits, where BWT was the original record data, and WWT and SMW of ewes were corrected by the constructed weight correction formula. Nevertheless, due to the small number of 6-month-old male sheep, all data were based on the original records at the predetermined time.

### Growth model

Logistic, Gompertz, and Von Bertalanffy models are the most growth curve fitting analysis to reflect the production performance of individual animals and their populations, therefor to predict their growth patterns.^8^^,^^9^ In the present study, three nonlinear animal growth curve models were exploited to fit the growth curves of crossbred sheep from 0 to 210 days. The growth model expressions and parameters are shown in Table 1, and comparative selection of the best fitting curve is based on the coefficient of determination R^2^.

**Table 1.** Growth curve model and characteristic parameter expressions.

Model	Function	Inflection point weight	Maximum daily gain
Logistic	$\text{W=}\frac{A}{1+Be^{\left(-Kt\right)}}$	A/2	Kw/2
Gompertz	$\text{W = }A\times e^{\text{(}-B\times e^{\left(-Kt\right)}\text{)}}$	A/e	Kw
Von Bertalanffy	$\text{W = }A\times {\left(1-Be^{\left(-Kt\right)}\right)}^{3}$	8 A/27	3*Kw*/2

W is the weight of *t* days, *A* is the limit growth amount, *K* is the instantaneous relative growth rate, *B* is the constant scale, *e* is a natural constant, t is the day age; *w* is the inflection point weight. The same as below.

### Genetic parameter estimation model

Based on the original data of early growth traits and the basis of previous research, sex (Sx), birth month (Bm), and Birth type (Number of Same Births, Bt) were selected as non-genetic factors.^10–12^ The F statistical test was carried out to investigate whether the effect of each factor in the fixed effects on weight traits at different growth stages of hybrid mutton sheep was significant or not, followed by highly significant fixed effects (*p* < 0.001) were added into the genetic parameter estimation model. After the models were evaluated and compared with Akaike information criterion (AIC), Bayesian information criterion (BIC) index and likelihood ratio test (LRT) test to explore the effects of maternal additive genetic effects and different data structures on the estimation of genetic parameters of hybrid sheep, we screen out the perfect animal model, which is suitable for the estimation of genetic parameters of economic traits in hybrid sheep, so as to estimate the final heritability of each trait. In this study, ASReml software (version.1.0.176) was used for analysis, two single-trait animal models were utilized to estimate the genetic parameters of economically important traits in hybrid sheep, and the model expressions were as follows:

(model 1) y=Xβ+Zα+e,

(model 2) y=Xβ+Zα+Fp+e,

where y is a vector of observations for early growth traits; β is a fixed effect; α is an individual additive genetic effect; p is a maternal additive genetic effect; e is a vector of residual effects; and X, Z, and F denote the structural matrices of nongenetic factors (fixed effects), individual additive genetic effects, and maternal additive genetic effects, respectively.

## Results

### SH, DH sheep growth curve fitting

#### SH, DH sheep growth curve model fitting analysis

**Figure 1.**
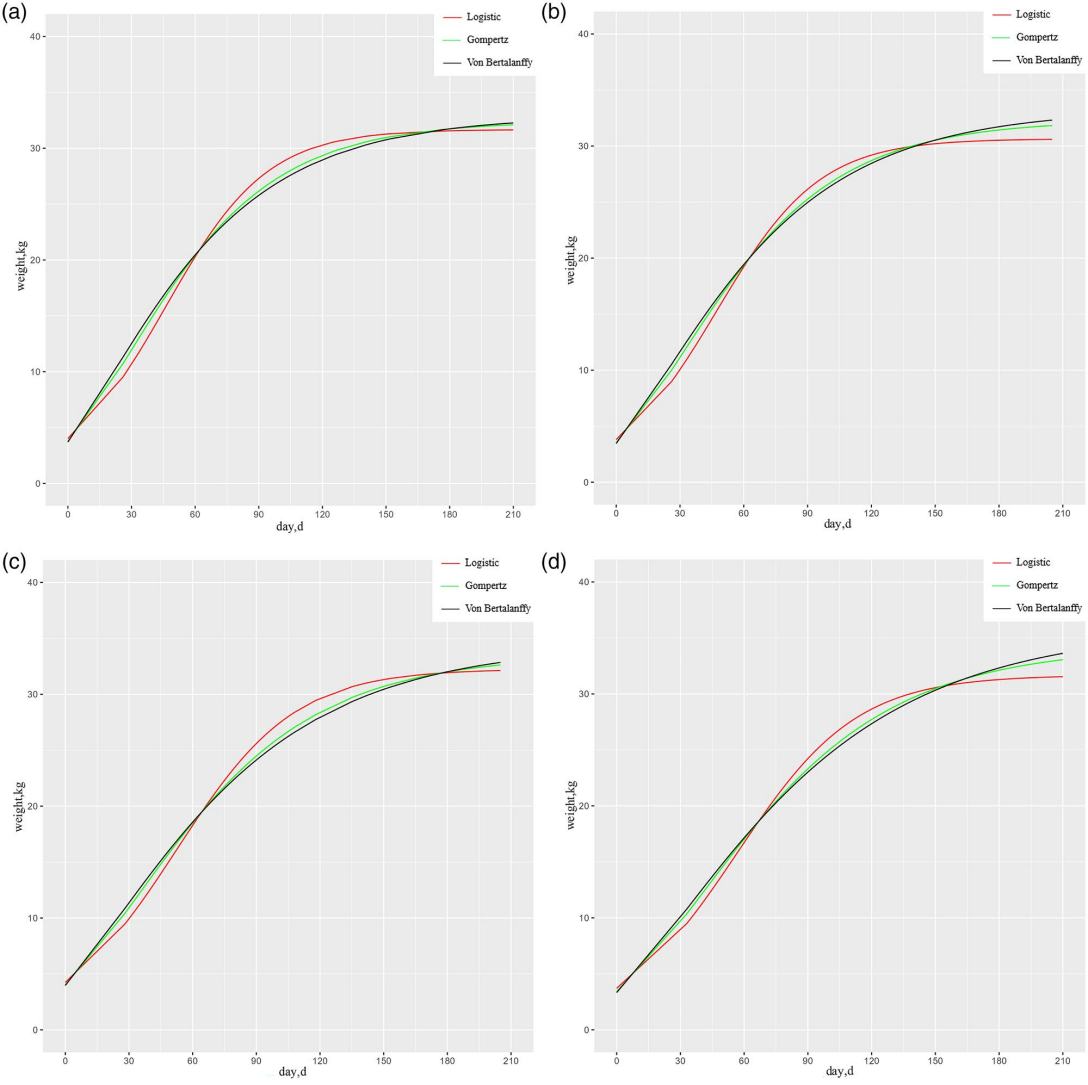
(a) Three model growth fitting curves of SH rams; (b) three model growth fitting curves of SH ewes; (c) three model growth fitting curves of DH rams; (d) three model growth fitting curves of DH ewes.

Logistic, Gompertz, and Von Bertalanffy growth models were contributed to establish the growth and developmental patterns of SH and DH sheep, the parameter estimation and fit analysis. As shown in Table 2, the fit of SH and DH sheep was above 0.900 in all three models, Gompertz, Logistic, and Von Bertalanffy. The Von Bertalanffy model had a high fit for both SH and DH sheep, with 0.917, 0.923, 0.925 and 0.918, respectively. Whereas, SH, DH ram and ewe growth fitting curves are shown in Figure 1, respectively.

**Table 2.** Fitting correlation coefficients of DH and SH sheep growth models.

Breed	Sex	Model	Parameters				Inflection point weight, kg	Maximum daily, g
			*A*	*B*	*K*	*R^2^*		
SH	Male	Logistic	31.675	6.887	0.042	0.911	15.838	330.215
		Gompertz	32.420	2.154	0.026	0.916	11.927	305.323
		Von Bertalanffy	32.881	0.517	0.021	0.917	9.742	306.885
	Female	Logistic	30.645	7.065	0.041	0.913	15.323	316.411
		Gompertz	32.306	2.214	0.024	0.921	11.885	289.985
		Von Bertalanffy	33.269	0.530	0.020	0.923	9.858	288.334
DH	Male	Logistic	32.259	6.606	0.036	0.919	16.129	289.523
		Gompertz	33.531	2.122	0.021	0.924	12.335	261.506
		Von Bertalanffy	34.491	0.514	0.017	0.925	10.219	259.066
	Female	Logistic	31.675	7.561	0.036	0.904	15.837	281.905
		Gompertz	34.240	2.307	0.019	0.915	12.596	250.664
		Von Bertalanffy	35.944	0.548	0.015	0.918	10.650	244.416

#### Comparison of measured and estimated body weights of SH and DH sheep

As shown in Tables 3 and 4, the three models estimated body weight at individual emerged the similar results compared with that of measured data. The measured body weights of SH and DH sheep at 60, 120 and 180 days were in better agreement with the estimated values.

**Table 3.** Comparison of measured and estimated values of body weight in SH and DH sheep.

Breed	Sex	Age, d	Measured value, kg	Model estimates, kg		
				Logistic	Gompertz	Von Bertalanffy
SH	Male	60	20.65 ± 3.923	20.251	20.394	20.430
		120	30.05 ± 5.754	30.276	29.342	28.945
		180	32.29 ± 4.355	31.556	31.731	31.730
	Female	60	19.65 ± 3.655	19.240	19.358	19.400
		120	28.93 ± 4.113	29.193	28.697	28.428
		180	33.26 ± 4.439	30.518	31.433	31.712
DH	Male	60	18.90 ± 4.263	18.262	18.502	18.574
		120	29.83 ± 5.604	29.624	28.384	27.956
		180	33.39 ± 5.608	31.929	32.000	32.014
	Female	60	17.60 ± 3.548	16.731	17.022	17.137
		120	27.89 ± 3.497	25.652	27.705	27.322
		180	33.10 ± 4.742	31.285	32.112	32.312

**Table 4.** Correlation between measured and estimated values of body weight in SH and DH sheep.

Breed	Sex	Age, d	Correlation coefficient		
			Logistic	Gompertz	Von Bertalanffy
SH	Male	60	0.981	0.988	0.989
		120	1.008	0.976	0.963
		180	0.977	0.983	0.983
	Female	60	0.979	0.985	0.987
		120	1.009	0.992	0.983
		180	0.918	0.945	0.953
DH	Male	60	0.966	0.979	0.983
		120	0.993	0.952	0.937
		180	0.956	0.958	0.959
	Female	60	0.951	0.967	0.974
		120	0.920	0.993	0.980
		180	0.945	0.970	0.976

### Corrected formula for body weight at 60 and 180 days

In regard to the construction of the correction formula, there will be some differences in the degree of accuracy due to the different ranges of the fitted data, therefore, the data with a coefficient of variation that less than 15% are selected for linear regression analysis, and then a more accurate correction formula will be constructed.

#### Corrected formula for weight at reaching 60 days

SH and DH ewes were selected from the range of 50–80 days and body weights of 12–25 kg. Table 5 showed the similar variation coefficients between body weight and day, indicated the similarity between variation degree in the determined body weights and day in these two groups. Meanwhile, considering the coefficients of variation of body weight and days of ewes in these two groups were less than 15%, which indicated that the degree of variation of measured body weight and days of SH and DH ewes was lower, and could be used for the subsequent study.

**Table 5.** The mean, standard deviation, and coefficient of variation of weight and age for each breed of ewes.

Breed	Weight			days		
	Mean, kg	Std, kg	CV, %	Mean, d	Std, d	CV, %
SH	19.58	2.349	12.00%	61.75	6.651	10.77%
DH	18.77	2.196	11.70%	61.77	7.027	11.38%

#### Correction formula for body weight of SH ewes and DH ewes reaching 60 days

The univariate linear regression model obtained by fitting was: y=0.151x+8.739 (y: denotes body weight, x: denotes day old) and the significance of the linear regression between day and body weight was tested. The analysis showed that there is a highly significant linear relationship between body weight and their age of SH ewes. so the linear regression equation between age and body weight can be utilized to construct the body weight correction formula by substituting x=60 into y=0.151x+8.739 to obtain y=17.799, by which it can be deduced that the body weight correction formula for SH ewes reaching the age of 60 days is as follows:$\begin{array}{l} \;W_{\text{calibration }60}=W_{\text{measured}}\times 17.799/[17.799+0.151\times (T_{\text{measured}}-\\ 60)]. \end{array}$

The univariate linear regression model obtained by fitting was: y=0.097x+11.769 (y: denotes body weight, x: denotes day old) and the significance of the linear regression between day and body weight was tested. The analysis showed that there is a highly significant linear relationship between body weight and their age of DH ewes, so that of the linear regression equation can be utilized to construct the body weight correction formula by substituting x=60 for y=0.097x+11.769 to obtain y=17.589, and the body weight correction formula for DH ewes reaching the 60th (weaning) day can be obtained by derivation as:$\begin{array}{l} W_{\text{calibration }60}=W_{\text{measured}}\times 17.589/[17.589+0.097\times (T_{\text{measured}}-\\ 60)]. \end{array}$

#### Corrected formula for weight at reaching 180 days

SH and DH ewes were selected from the age range of 150–210 days and body weights of 20–45 kg. Table 6 showed the similar variation coefficients between body weight and day, indicated the similarity between variation degree in the determined body weights and day in these two groups. Meanwhile, considering the coefficients of variation of body weight and days of ewes in these two groups were less than 15%, which indicated that the degree of variation of measured body weight and day old of SH and DH ewes was lower, and could be used for the subsequent study.

**Table 6.** The mean, standard deviation, and coefficient of variation of weight and age for each breed of ewes.

Breed	Weight			Days		
	Mean, kg	Std, kg	CV, %	Mean, d	Std, d	CV, d
SH	32.52	4.179	12.85%	185.59	15.082	8.13%
DH	33.84	4.233	12.50%	182.33	15.063	8.26%

#### Correction formula for body weight of SH ewes reaching 180 days

The univariate linear regression model obtained by fitting was: y=0.056x+20.219 (y: denotes body weight, x: denotes day old) and the significance of the linear regression between day and body weight was tested. The analysis showed that there is a highly significant linear relationship between body weight and their age of SH ewes, so that of the linear regression equation between day and body weight can be utilized to construct the body weight correction formula, substituting x=180 for y=0.056x+20.219 to obtain y=30.299, and the body weight correction formula for SH ewes reaching 180 days can be obtained through derivation as follows:$\begin{array}{l} \;W_{\text{calibration }180}=W_{\text{measured}}\times 30.299/[30.299+0.056\times (T_{\text{measured}}\\ -180)]. \end{array}$

The univariate linear regression model obtained by fitting was: y=0.109x+11.049 (y: denotes body weight, x: denotes day old) and the linear regression between day and body weight was tested. The analysis showed that there is a highly significant linear relationship between body weight and their age of DH ewes, so the linear regression equation between age and body weight can be used to construct the body weight correction formula, substituting x=180 for y=0.109x+11.049 to obtain y=30.669, and through the derivation of the body weight correction formula for DH ewes reaching the age of 180 days can be derived as follows:$\begin{array}{l} \;W_{\text{calibration }180}=W_{\text{measured}}\times 30.669/[30.669+0.109\times (T_{\text{measured}}\\ -180)]. \end{array}$

#### Comparison of body weight measurements and corrected values at 60 and 180 days in DH and SH ewes

The correlation coefficient test was performed on five DH and SH ewes randomly selected from the group possessing body weights at 60 and 180 days, respectively, and the results were shown in Tables 7 and 8. The measured values of body weights of 60 and 180 days of DH and SH ewes were close to the calibrated values, and the degree of agreement was high.

**Table 7.** Weights measured at 60 days for DH and SH ewes were compared with corrected values.

Age, d	Measured value, kg	Breed	Weight measurements at 60 days, kg	Weight correction at 60 days, kg	Correlation coefficient
54	18.68	SH	20.19	19.68	0.975
63	18.50		18.41	18.04	0.980
67	19.53		18.85	18.43	0.978
70	19.62		18.27	18.08	0.990
77	23.38		20.58	20.43	0.993
51	14.87	DH	15.76	15.65	0.993
57	18.56		19.16	18.87	0.985
61	20.46		20.57	20.35	0.989
62	16.57		16.46	16.39	0.996
75	23.59		21.90	21.79	0.995

**Table 8.** Weights measured at 180 days for SH and DH ewes were compared with corrected values.

Age, d	Measured value, kg	Breed	Weight measurements at 180 days, kg	Weight correction at 180 days, kg	Correlation coefficient
160	20.50	SH	22.00	21.29	0.968
178	22.62		23.19	22.70	0.979
193	30.36		29.98	29.65	0.989
198	41.50		40.56	40.16	0.990
205	39.85		38.90	38.09	0.979
157	23.30	DH	25.50	25.37	0.995
170	22.50		23.45	23.33	0.995
189	33.35		32.78	32.32	0.986
196	35.50		33.86	33.59	0.992
207	29.25		26.96	26.69	0.990

### Effect of non-genetic factors on early growth traits in DH and SH sheep

#### Descriptive statistics of body weight at different growth stages of DH and SH sheep

Using the constructed formula to correct the recorded measurement data, there were 9542 of DH and SH sheep, of which the mean values of BWT, WWT and SMW were 3.93 kg, 18.63 kg and 33.90 kg for DH sheep, and 3.84 kg, 19.04 kg and 33.23 kg for SH sheep. The coefficients of variation ranged from 18% to 29%. 18%∼29%, which belonged to medium variance traits. As shown in Table 9.

**Table 9.** Descriptive statistics of early growth traits.

Traits	DH sheep			SH sheep		
	BWT	WWT	SMW	BWT	WWT	SMW
Numbers	5,761	3,519	846	3,781	2,256	619
Minimum	1.00	10.62	17.17	1.00	9.77	17.61
Median	3.60	17.25	29.40	3.50	17.08	27.68
Maximum	7.90	28.28	48.91	6.90	29.34	46.40
Means, kg	3.93	18.63	33.90	3.84	19.04	33.23
Std, kg	1.07	3.56	5.47	0.96	3.65	5.54
C.V., %	0.29	0.20	0.18	0.27	0.21	0.20

#### Significance of different non-genetic factors on early growth traits in DH and SH sheep

The significance of Sx, Bm and Bt on BWT, WWT, and SMW was tested by R language, respectively. As shown in Table 10, the results showed that Tb and Bm exhibited highly significant effects (*p* < 0.001) on BWT, WWT, and SMW in DH and SH sheep, and should be placed in the model as a fixed effect; and Sx had highly significant effects (*p* < 0.001) on BWT, WWT, and SMW in DH and SH sheep.

**Table 10.** Effects of non-genetic factors on early growth traits.

Breed	Growth traits	Traits	Non-genetic factors		
			Sx	Bt	Bm
DH	BWT	DF	1	4	11
		F-value	94.44	1775.3	99.90
		P-value	2.2e-16***	2.2e-16***	2.2e-16***
	WWT	DF	1	4	11
		F-value	21.7	131.5	45.1
		P-value	3.144e-6***	2.2e-16***	4.708e-6***
	SMW	DF	1	3	11
		F-value	0.6	65.8	105.1
		P-value	0.4331	3.331e-14***	2.2e-16***
SH	BWT	DF	1	5	11
		F-value	54.85	822.36	85.3
		P-value	1.303e-13***	2.2e-16***	1.38e-13***
	WWT	DF	1	5	10
		F-value	12.08	76.61	147.07
		P value	0.510e-3***	4.33e-15***	2.2e-16***
	SMW	DF	1	3	11
		F-value	4.77	20.13	91.05
		P-value	0.029*	0.160e-3***	1.021e-14***

*p < 0.05. **p < 0.01. ***p < 0.001.

#### Estimation of variance components of body weight at different growth stages in DH and SH sheep

The results of Table 11 showed the different models for estimating the variance components of body weight at different growth stages of DH and SH sheep, including the residual effect (${\sigma e}^{2}/{\sigma y}^{2}$) variance components of BWT and WWT of DH and SH sheep were larger than those of Model 2 and also than that of Model 1; and the proportion of the variance components of the individual permanent environment effect (${\sigma p}^{2}/{\sigma y}^{2}$) in Model 2 decreased with increasing age.

**Table 11.** Weight variance components of DH and SH sheep at different growth stages estimated by different models.

Breed	Growth traits	Model	σy^2^	σa^2^	σp^2^	σe^2^	σa^2^/σy^2^	σp^2^/σy^2^	σe^2^/σy^2^
DH	BWT	1	1.117	1.062		1.190 × 10^–5^	0.951		1.065 × 10^–5^
		2	1.117	0.066	0.300	0.467	0.059	0.269	0.418
	WWT	1	8.235	6.055		2.939	0.735		0.357
		2	8.235	0.127	1.896	5.631	0.015	0.230	0.684
	SMW	1	28.287	2.257		21.720	0.080		0.768
		2	28.287	0.761	6.384	16.688	0.027	0.226	0.590
SH	BWT	1	0.916	0.918		1.040 × 10^–7^	1.002		1.135 × 10^–7^
		2	0.916	0.012	0.273	0.428	0.013	0.298	0.467
	WWT	1	13.342	9.966		4.066	0.747		0.305
		2	13.342	0.275	2.803	8.861	0.021	0.210	0.664
	SMW	1	30.626	2.743		23.056	0.090		0.753
		2	30.626	1.346	3.127	21.121	0.044	0.102	0.690

σy^2^: phenotypic variance; σa^2^: direct additive genetic variance; σp^2^: maternal additive genetic variance; σe^2^: residual variance; σa^2^/σy^2^: direct additive genetic effect; σp^2^/σy^2^: maternal additive effect; σe^2^/σy^2^: residual effect; the blank indicates that there is no such effect in the model.

#### Evaluation of different models using AIC and BIC

The results are showed in Table 12. The results of AIC and BIC model 1 for body weight traits at different growth stages in DH and SH sheep were greater than that of Model 2, which scored better overall.

**Table 12.** Standard values of AIC and BIC information in different models of body weight at different growth stages.

Breed	Growth traits	Model	AIC	BIC
DH	BWT	1	4,184.561	4,197.857
		2	4,130.978	4,150.922
	WWT	1	7,645.041	7,656.693
		2	7,627.557	7,645.036
	SMW	1	3,437.184	3,446.578
		2	3,430.563	3,444.654
SH	BWT	1	2,119.635	2,132.011
		2	2,096.792	2,115.354
	WWT	1	7,595.268	7,606.649
		2	7,577.422	7,594.493
	SMW	1	2,566.377	2,575.147
		2	2,566.294	2,574.450

#### Comparison of different models using LRT

LRT and chi-square test difference significance results accomplished from the comparison of different random effects models are listed in Table 13. LRT were conducted for each trait using Model 1 vs Model 2. The results showed that the difference between Model 1 and Model 2 calculations was highly significant in the estimation of genetic parameters for early growth traits in DH and SH sheep.

**Table 13.** Chi-square test results of growth traits by different animal models.

Traits	DH sheep			SH sheep		
	BWT	WWT	SMW	BWT	WWT	SMW
LRT	55.584	19.483	8.621	24.844	19.846	8.083
Chi	4.48e-14***	5.08e-6***	1.66e-3**	3.109e-7***	4.197e-6***	1.78e-3**

#### Heritability of early growth traits in DH, SH sheep

As shown in Table 14, our study showed that the primordial heritability of BWT, WWT, and SMW is ranged from 0.062 to 0.106 for DH, SH sheep, and the maternal effect heritability is ranged from 0.071 to 0.186.

**Table 14.** Heritability and standard error early growth traits of DH and SH sheep.

Breed	Growth traits	Heritability ± SE	Maternal effect heritability ± SE
DH	BWT	0.104 ± 0.098	0.186 ± 0.020
	WWT	0.093 ± 0.097	0.115 ± 0.022
	SMW	0.062 ± 0.025	0.071 ± 0.049
SH	BWT	0.106 ± 0.113	0.177 ± 0.009
	WWT	0.080 ± 0.083	0.108 ± 0.040
	SMW	0.065 ± 0.094	0.081 ± 0.072

## Discussion

It is well-known that, animal growth generally exhibited the non-linear characteristics. Fortunately, researchs on account of the curve fitting analysis of growth and development provided a powerful guidance on seed selection and breeding work in actual production.^13^ Thus, non-linear mathematical models can be used to describe the growth patterns of animals, therefore obtaining descriptive information and predictions on growth and development. Taken advantage of specific fitting models contraposed to the different livestock species and sex so that more accurately estimate their growth. While model fit can be judged to filter out the optimal model, it is necessary to select the best model based on the correction coefficients as well.^14^ As we known, hence the body weight regularly changes along with the aging growth, and as a digital representation, the growth curve model can perfectly understand the overall development trend, therefore predict the body weight, which can guide the production practice, rationalize the arrangement of feeding management, and thus improve the economic efficiency. The fitting degree of all three models in the present study was above 0.900, combined with the high the agreement between measured and predicted values, indicated that all three models were able to fit the growth and development of crossbred sheep consummately. Among them, Von Bertalanffy nonlinear model fitting the weight growth curves of SH and DH rams and ewes better than that of those two models. Our research is in agreement with the previous findings, showed that Von Bertalanffy model fitted the growth curve of Awassi sheep,^15^ and Norduz sheep,^16^ therefore Von Bertalanffy model provided a best model that reflect the growth performance. Inconsistent with the study of McManus *et al.*^17^ who found that the Logistic model showed a better fit degree by fitting growth curves of Bergamasca sheep using the Brody, Richards, and Logistic model, accompanied with Nursen *et al.*^18^ who concluded that the Gompertz model was the best model for describing the growth of Kivircik sheep by fitting and analyzing the growth curves of Kivircik sheep. This contradiction might be due to differences in varieties, climatic environments, and other factors. Taken together, different models should be utilized to address the variability exhibited different varieties, and the best growth curve fitting model should ultimately be selected through a comprehensive analysis of its goodness-of-fit, variance, and other aspects.

Considering the impossiblity to acquire the body weight of each sheep at the desired time in practical production, especially in large-scale house-feeding groups, so a certain correction method to correct the actual measurement value of each sheep to the same age, which can be used for the subsequent genetic evaluation, the calculation of the comprehensive breeding index has been become an urgent issue. In the case of construction of the calibration formula, due to the different ranges of fitted data, there will be some differences in the degree of accuracy, therefore, selection the fitted data with coefficients of variation less than 15%, will achieve a high degree of accuracy of the calibration. According to the results of our analysis, it can be seen that there is a highly significant linear relationship between body weight and age in different breeds of sheep, and these significant correlations can be utilized in the selection of traits to achieve the purpose of selecting the target traits. Taken together, we constructed the body weight correction formula through a linear regression equation at the age of 60, 180 days of SH and DH ewes can be undoubtedly used in actual production. The slopes of our four linear regression equations were 0.151 (SH ewes at 60 days), 0.097 (DH ewes at 60 days), 0.056 (SH ewes at 180 days) and 0.109 (DH ewes at 180 days), which proved that at 60 days SH sheep grow faster than DH sheep. This is corroborates with the maturity rate represented by the parameter *k* in the fitted curve, where the higher the value of *k* reflect the faster the early growth and development of the animal.^9^ In the present study, we found that the growth rate of SH sheep before reaching 30 kg was faster than that of DH sheep, indicating that SH sheep exhibited more outstanding early growth and development performance than that of DH sheep. Therefore, the corresponding weight correction formula should be applied according to different breeds and growth stages in actual production. Interestingly, our correction formula was mainly focused on the ages of 50–80 and 150–210 days, and the body weight measurements of DH and SH ewes at weaning age and 180 days exposed a good agreement with the corrected values, especially. Consequently, the formulas constructed in this study not only showed a strong applicability, but demonstrated a splendid accuracy also.

One after another reports on non-genetic factors in sheep revealed the factors affecting the production performance, especially the birth month, type of birth, sex, and flock, *etc*. Combined with the positive control of these factors, people zealously estimated the genetic parameters, develop the rational breeding programs, as well as establish the genetic evaluation models to eliminate to negative non-genetic factors to accelerate the breeding process.^19–21^ Indeed, reasonable analytical models play a pivotal role in estimation of genetic parameters of quantitative traits. In terms of genetic parameter estimation models, the genetic evaluation of individual animals and genetic parameter estimation methods, which is based on the mixed-model system of equations (MMSE) theory, have replaced the traditional selection index method and sibling or parent-child regression analyses. Honestly, the genetic parameter estimation methods contributed more accurate genetic evaluation of individuals and more reliable estimation of genetic parameters, and have substantially increased the speed of genetic improvement of some economically important traits. As we known, different data structures, fixed effects and random effects in animal models affected the estimation of genetic parameters, so estimating genetic parameters of different populations should be based on the actual data structure and field conditions to choose the appropriate animal model.^22^ The variance of the breeding values is belong to the additive genetic variance, is the main cause of similarity between relatives, and is thus the primary determinant of the measurable genetic characteristics of a population and of the population’s response to selection. While mammals, due to the presence of long-term maternal dependence, the early growth traits are controlled not only by the direct additive genetic effects, but also by maternal effects.^23^^,^^24^ Recently developed statistical software, ASReml, fitted the linear mixed models using the linear great likelihood method, which is widely used in plant and animal quantitative genetics and breeding research.^25^ Using the average information algorithm and sparse matrices, it not only exhibited a high analytical efficiency for mixed models, but also achieves excellent results in the analysis of generalized linear models and general linear models. Its superiority is mainly reflected in dealing with the balanced and unbalanced data during multiyear, making full use of inter-individual genealogical information to establish kinship matrices for accurate estimation of genetic parameters and prediction of breeding values, as well as the G-structure and R-structure in the formula of the mixed model, and so on. In this study, the genetic parameters of economically important traits in crossbred mutton sheep were estimated using a single-trait animal model based on ASReml, which considered all the kinship relationships, utilized as many as possible genealogical information, maximized minimized the bias due to nonrandom mating and selection of females, as well as certain individual’s toward. Our research showed that Bt and Bm had highly significant effects (*p* < 0.001) on BWT, WWT, and SMW in DH and SH sheep, and Sx had highly significant effects (*p* < 0.001) on BWT and WWT in DH and SH sheep, and that these nongenetic factors could be considered as fixed effects in the genetic evaluation model. In agreement with our study, previous reports found the influence factors of growth, wool production, early growth, and reproduction traits by fixed effects analysis were affected by birth type, sex, pedigree, flock, age of the individual, and age of the ewe.^26–30^ Collectively, these findings suggested that the influence of non-genetic factors such as sex, birth month, and birth type should be taken into account when estimating genetic parameters.

Taken account of the excellence of model fitting is evaluated by the AIC and BIC. The formula for the AIC index is: AIC = 2*k*-2ln(*L*), where *L* is the maximum likelihood function and *k* is the number of parameters to be estimated,^31^ increasing the number of parameters in the model improves the excellence of the fit but tends to result in overfitting, so the AIC improving fit while it declined an function of the number of parameters. The AIC value is the smallest one among the candidate models for preseted data. On the other hand, BIC is an informative criterion for selecting model among a limited models, the formula: BIC = ln(n)*k*-2ln(*L*), where *L* is the maximum likelihood function, *k* is the number of parameters in the model, and n is the number of samples. The lower the value of BIC reflect the better the model fit. Both BIC and AIC address the problem of overfitting by introducing a penalty term to solve the overfitting problem, and the penalty of BIC is greater than that of AIC.^32^ Moreover, the LRT is a statistical test of the goodness of fit between the two models; a relatively full model is compared to a reduced model to determine importance of a parameter that gives rise to increase in log-likelihood. That is to say, the full model must differ from the reduced model only by the addition of one or more parameters. In other words, LRT explains whether it is good to add a parameter to a model or not. The test begins with a comparison of the likelihood scores of the two models, after which follows a chi-squared distribution with degrees of freedom equal to the difference in dimensionality of the models.^33^ In this study, the maternal additive genetic effect was added to model 2 compared with model 1, and in the estimation of variance components, the maternal additive genetic effect accounted for a much higher proportion than the individual additive genetic effect and the residual effect, which indicated that the maternal additive genetic effect was significant in the estimation of the genetic parameters of body weight for DH and SH sheep. It was found that the AIC and BIC results of model 1 were greater than those of model 2 for different growth stages of DH and SH sheep, and the LRT test showed that these two models differed significantly, which indicated that model 2 was the best model for the estimation of genetic parameters of body weight in DH and SH sheep. This result is consistent with the findings of Hızlı *et al.* who found that the effects including maternal genetics or maternal permanent environment effects were significant in parameter estimation in the genetic evaluation on body weights in Awassi sheep.^34^

Heritability is the cumulative variable portion of the phenotype resulting from the cumulative effect of genes, and is also the portion of the trait that can be inherited and fixed in the transmission of the trait from parents to offsprings. The most important role of heritability is to estimate the reliability of phenotypic values of breeding values.^35^ Jalil-Sarghale *et al.*^36^ estimated genetic parameters for early growth traits in Baluchi sheep with heritability of 0.062, 0.12, 0.08 for birth weight, weaning weight, average daily gain before weaning and 0.09, 0.04, 0.03 for maternal effect, respectively; Jafaroghli *et al.*^37^ estimated heritability of 0.07 for birth weight and 0.18 for maternal genetic effect; heritability of 0.09 for weaning weight and 0.06 for maternal genetic effect; and heritability of 0.08 for average daily weight gain prior to weaning in Moghani sheep. In the present study, the heritabilities of DH and SH sheep for birth weight, weaning weight, and six months weight was 0.1041, 0.0932, 0.0616, 0.1063, 0.0798, and 0.0649, respectively, and maternal heritabilities were 0.1859, 0.1146, 0.0709, 0.1771, 0.1078, and 0.0806, respectively. In conclusion, the results of this study are within the range of the heritability of similar sheep breeds, like Baluchi sheep, Sardi sheep, and Moghani sheep, and it was found that the differences in the heritability was not only caused by the breeds and environments, but also affected by a variety of other factors, such as data structure, model construction, application software, and computational methods.

## Conclusions

In summary, the present study, it was found that both of DH and SH sheep exhibited the potential for rapid early growth and development through fitting the growth curves, SH sheep grow faster than DH sheep in the early stage, but during the long growth period DH sheep grow faster than SH sheep in the later stage. These findings gave us outstanding idea for breeding work. In addition, Sx, Bt and Bm non-genetic factors extremely affected the early growth traits of DH and SH sheep, and the maternal effect should be taken into consideration for the purpose of improving accuracy in parameter estimations and therefore increasing the success of breeding programs. Taken together, the results of this study not only provided a scientific basis for the estimation of early growth, development patterns and genetic parameters of DH and SH sheep, but also afforded a theoretical basis for accelerating the breeding process of new breeds of specialized housed-feeding mutton sheep.
